# Neither carrots nor sticks? Challenges surrounding data sharing from the perspective of research funding agencies—A qualitative expert interview study

**DOI:** 10.1371/journal.pone.0273259

**Published:** 2022-09-07

**Authors:** Michael Anger, Christian Wendelborn, Eva C. Winkler, Christoph Schickhardt

**Affiliations:** 1 Section for Translational Medical Ethics, National Center for Tumor Diseases (NCT) Heidelberg, German Cancer Research Center (DKFZ), Heidelberg, Germany; 2 Section for Translational Medical Ethics/Department of Medical Oncology, National Center for Tumor Diseases (NCT) Heidelberg, University Hospital Heidelberg, Heidelberg, Germany; University of Texas Southwestern Medical Center at Dallas, UNITED STATES

## Abstract

**Background:**

Data Sharing is widely recognised as crucial for accelerating scientific research and improving its quality. However, data sharing is still not a common practice. Funding agencies tend to facilitate the sharing of research data by both providing incentives and requiring data sharing as part of their policies and conditions for awarding grants. The goal of our article is to answer the following question: What challenges do international funding agencies see when it comes to their own efforts to foster and implement data sharing through their policies?

**Methods:**

We conducted a series of sixteen guideline-based expert interviews with representatives of leading international funding agencies. As contact persons for open science at their respective agencies, they offered their perspectives and experiences concerning their organisations’ data sharing policies. We performed a qualitative content analysis of the interviews and categorised the challenges perceived by funding agencies.

**Results:**

We identify and illustrate six challenges surrounding data sharing policies as perceived by leading funding agencies: The design of clear policies, monitoring of compliance, sanctions for non-compliance, incentives, support, and limitations for funders’ own capabilities. However, our interviews also show how funders approach potential solutions to overcome these challenges, for example by coordinating with other agencies or adjusting grant evaluation metrics to incentivise data sharing.

**Discussion and conclusion:**

Our interviews point to existing flaws in funders’ data sharing policies, such as a lack of clarity, a lack of monitoring of funded researchers’ data sharing behaviour, and a lack of incentives. A number of agencies could suggest potential solutions but often struggle with the overall complexity of data sharing and the implementation of these measures. Funders cannot solve each challenge by themselves, but they can play an active role and lead joint efforts towards a culture of data sharing.

## 1 Introduction

The sharing of research data is widely perceived to be crucial for accelerating scientific progress [1, 2]. Data Sharing promotes research efficiency and reliability, new research approaches and collaborations, as well as the reproducibility of scientific results [3–5]. Moreover, it not only adds to the quality of science but also benefits society as a whole [6]. Most stakeholders within the scientific community consider data sharing to be a part of good scientific practice and research integrity [7–10]. However, data sharing is still not common practice [11–17]. Several prevalent challenges and conflicts regarding data sharing point to a rocky road when it comes to its implementation. The existing hurdles for researchers to share data are manifold and include ethical, legal, economic, or motivational barriers [18–24]. Researchers often lack incentives to share data, are unaware of the necessary procedures, or even feel the need to withhold data to advance their careers [1, 18, 25, 26].

This discrepancy between ideals and practices and the reported challenges for data sharing raise two initial questions: First, what can be done to close this gap and promote the practice of data sharing? Second, who can take a leading role in these efforts? Many believe that research funding agencies can play a central role in promoting data sharing, primarily by implementing data sharing policies [16, 24, 27–33]. Since they provide funding for research, they are in a powerful position to promote data sharing. Moreover, if they spend public money, they might even have a moral responsibility to the general public to promote or require data sharing in order to maximise the scientific and public benefit of their funding [25, 34, 35]. Several agencies have already implemented data sharing policies for funded research projects, but these are still at varying stages of development [30]. While a number of studies identifies data sharing policies as important guidelines for researchers [11, 36–38], research on the implementation and effects of data sharing policies also points to prevalent problems and room for improvement [18, 30, 33, 36–40]. However, there is little knowledge about how funding agencies themselves perceive their own role, their capabilities, and their limits when it comes to promoting data sharing. As such, the practical perspectives and experiences of funding agencies are still underexplored in research. Given the body of literature on the general challenges surrounding data sharing, the particular challenges for funding agencies regarding the design, implementation, or enforcement of their data sharing policies deserve more attention.

Drawing on a series of guideline-based interviews with contact persons for open science at international funding organisations, we aim to answer the following research question: *which challenges do international funding agencies perceive when it comes to their own efforts to foster and implement data sharing via their policies*? For this purpose, we use the terms “data sharing policies”, or “policies” of funders in a broad sense, referring to data sharing policies as a framework for grant conditions, instructions, incentives, evaluation, and monitoring criteria regarding data management and sharing. After laying out our socio-empirical materials and methods (chapter Two) we illustrate selected results from the interviews (chapter Three). Based on our empirical data, we categorise our findings into a set of six broader challenges expressed by funders in the interviews: The design of clear policies (I), monitoring of compliance with these policies (II), sanctions for non-compliance (III), providing incentives (IV), offering support (V), and limits to funders’ scope of action (VI). For each of these challenges, we highlight several aspects and manifestations exemplified by passages from our interviews. We also address perceived and intended solutions by funding agencies insofar as they were reported in the interviews alongside these challenges. We close with a discussion of our findings (chapter Four).

## 2 Methods

This article is embedded in an interdisciplinary research project that investigates the question of how public funding agencies could and should design their funding policies and grant conditions to encourage funded scientists to share their research data with the scientific community.

### Sample selection

**Fig 1:**
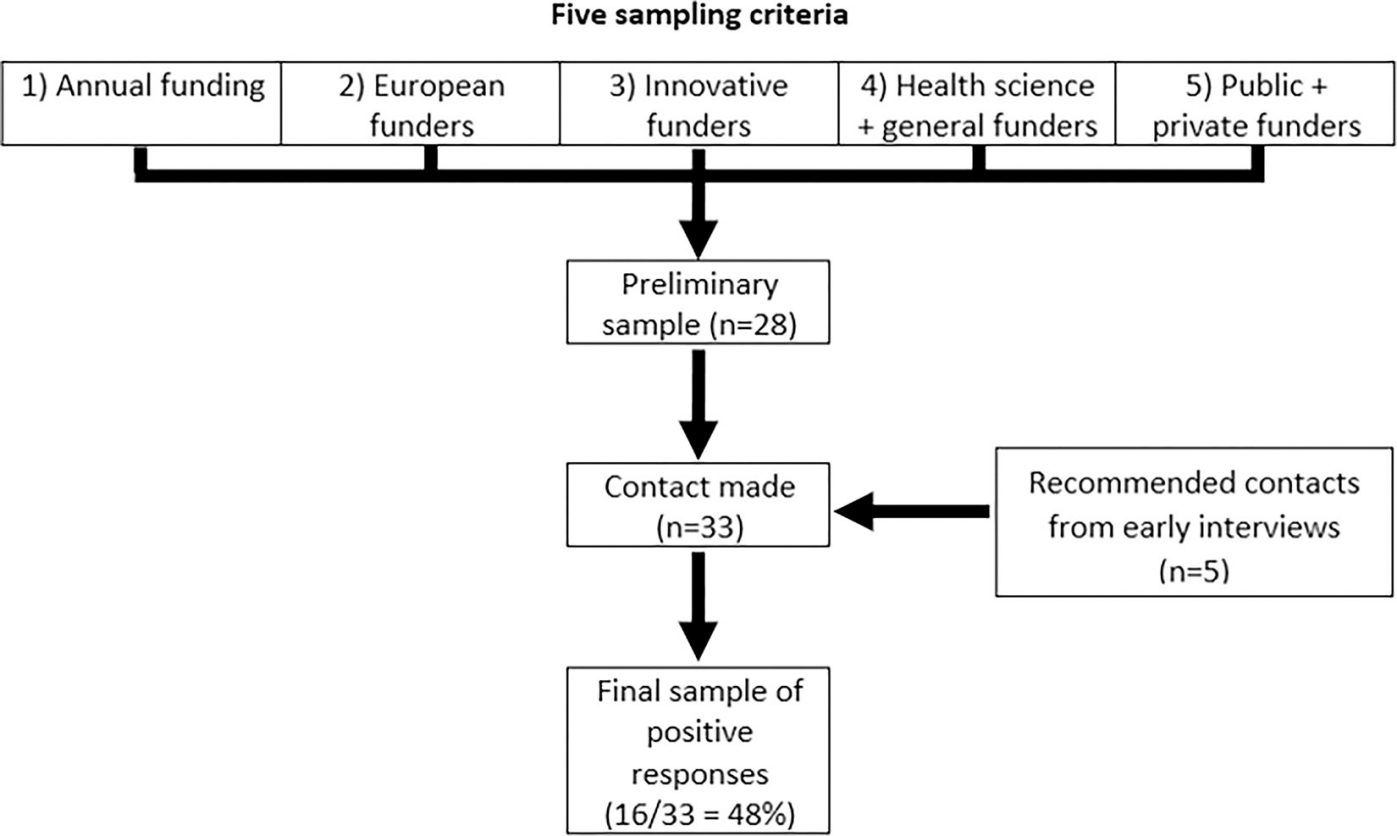
Sampling procedure.

Our sampling process included five criteria. First, the amount of funding, using the work of Viergiver and Hendriks [41, 42] to gain an overview of the largest funders in the field of health and life sciences in terms of annual spending. We opted for this particular field of research because funders and researchers in this area are considered to be rather advanced in their data sharing practices [24, 43, 44]. Second, some European funding agencies, which are leading national agencies in their respective countries, were added to the list. This allowed us to investigate a potential collaboration of funding agencies and convergence of policies within Europe, as several agencies are connected via funder networks such as Science Europe [45]. Third, we reviewed data sharing policies available on funders’ websites to identify agencies that have policies on data sharing, and then primarily contacted advanced and innovative agencies with detailed and elaborate policy documents. At a later stage, we also asked early interviewees about which other agencies they deemed exemplary or progressive and updated our sample accordingly. Fourth, we expected significant differences between funders specialised in the funding of health and life science research and funders invested in a number of different disciplines. The anticipated challenge of addressing a spectrum of scientific fields with policies led us include both kinds of agencies. Finally, we included some private funders to check for potential deviations from public funders. Our sampling criteria can be summed up in the following sampling procedure (Fig 1).

Overall, we contacted 33 funding agencies from Europe and North America, 27 of which are public and six of which are private. After approaching their respective Data Sharing, Open Research Data, Open Science or Open Access departments via email (with up to two reminders), our final sample included 16 funding agencies available for interviews (48% positive response rate). In total, we interviewed funders from ten different countries, six from the Anglosphere and ten from continental Europe. Seven of them specialise in health and life sciences, while nine of them fund research from a spectrum of different scientific fields. The vast majority of them are public funding agencies. Of the 17 unsuccessful interview requests, three agencies explicitly declined our request and 14 did not respond at all. To reduce the risk of any potential re-identification of the 16 interviewees and to ensure their anonymity, we assigned three different pseudonyms to each interview, leading to a list of 48 ciphers (Interview 01–48). This approach of using multiple ciphers for each interview is the most suitable way for us to cite passages from the interviews in the results section and maximise the scientific value of the interviews, while at the same time protecting the anonymity of the interviewees. It is particularly important because some interviewees made critical statements about their organisations’ efforts and strategies.

### Data collection

All interviews were conducted from November 2020 until May 2021 by video call, ranging in duration from 43 to 75 minutes. In order to perform a comparative content analysis at a later stage, the interviews followed a structured interview guideline (S1 Appendix). The guideline was designed based on our assessment of the literature on data sharing, an initial exploration of funding agencies’ data sharing policy documents and interdisciplinary discussions. The interviewees are contact persons for open science at their respective funding agencies and have job profiles such as (open science) policy advisors, (senior) science advisors or heads of open research. However, despite having provided insights into their organisations’ approaches, they do not necessarily offer the official position of their organisations. Nonetheless, as is common for the method of expert interviews, “experts” are not just deemed as particularly knowledgeable but are also “ambassadors” of their respective field to a certain degree [46]. Thirteen of the interviews were one-on-one conversations, while three interviewees asked to be joined by another colleague. With a few exceptions, all interviews were conducted in English. For the purpose of this article, the authors jointly translated passages from interviews conducted in other languages. After each interview, the recorded audio files were encrypted, anonymised, and transcribed, using the transcription rules defined in our transcription and coding guideline (S2 Appendix) and the software MAXQDA 2020 [47]. All interviews were transcribed by trained student assistants and crosschecked by the authors.

### Data preparation and analysis

To code the interview material, we used a specific method of qualitative content analysis [48]. First, we created a codebook to define a set of rules for the coding process based on an initial examination with the data. We drew on the project’s research questions, our interview guideline, and supplemental discussions to deductively develop preliminary major categories. The developed categories were defined and exemplified in the codebook, leading to a first draft of the category system. This was followed by an initial round of coding with the main categories using MAXQDA 2020, which served two purposes: first, to check the adequacy of the main categories. Second, it also allowed for a systematic review of the material according to the main categories, which helped identify patterns and peculiarities. This approach, in turn, led us to inductively develop relevant subcategories from the empirical material, culminating in a final category system (S3 Appendix) and its application to the interview data in a second round of coding. The entire coding process was conducted by a postdoctoral research associate and a research assistant, both trained sociologists. Based on our final version of the codebook, we jointly compared and reanalysed all the coding to reach a consensus [49]. In a final step, we analysed the content to determine our qualitative data from ethical and sociological perspectives, which led us to identify the six commonly perceived challenges in our interviews.

### Ethics statement

Informed consent was obtained for each interview. Every interviewee received detailed written information about the study and the procedure several days prior to the interview. Before starting the interview, each interviewee was offered additional explanations and given the opportunity to ask questions. Assessment and approval by the Ethics Commission of the Heidelberg University Hospital were not necessary for this study because neither patients nor physicians were involved. Data processing is officially registered with and approved by the data protection office of the German Cancer Research Center, which also approved the use of oral consent for the interviews (registration no. D120-P989). Oral consent was chosen over written consent for three reasons: First, all interviews were conducted by video call. Second, we reduced the amount of additional sensitive data by having both the consent and the audio data in one encrypted file. Third, all interviewees explicitly agreed to giving consent orally, which also reduced bureaucracy for them. Prior to obtaining oral consent for each interview, all interviewees were explicitly asked to agree to the start of the recording. All data processing was conducted in accordance with the European Union General Data Protection Regulation (GDPR).

## 3 Results

Our interviews with open science contacts at funding agencies regarding their experiences and perspectives on their own funding policies revealed some recurring patterns. Hereinafter, we categorise these patterns into six challenges perceived by funding agencies and illustrate them with quotes from the interviews. In doing so, we describe a range of related barriers and hurdles associated with these challenges, as well as funders’ approaches to overcome them. While we attempt to distinguish between challenges and solution approaches, some solutions themselves are also challenging to implement, making it sometimes difficult to draw an exact line between them. Please bear in mind that not all of our interviewees are native English speakers and even native speakers occasionally make linguistic mistakes in interview situations. We confined ourselves to redacting only major linguistic errors in order to strike a balance between the two important aspects of readability and authenticity of the interview quotes.

### Challenge I: Design of clear data sharing policies and concrete requirements

Several funders reported problems in designing and explaining their data sharing requirements. Some funders evaluated their own data sharing policies and requirements as vague and not sufficiently clear. They reported a lack of clarity about what exactly is required of researchers and saw a need for more detailed explication of their own expectations and requirements (e.g., when and how to share or what type of repositories to use).

*“[…] the recommendation was too soft*. *It’s a bit blurred what we are really asking for*. *And that has led to some confusion and I also believe that […] if we had been a bit more*, *not strict*, *but clearer in our communication*, *maybe there wouldn’t be so many different data management plans being developed in different areas and maybe it would be a bit more coherent”* **(Interview 47)**

Some funders reported the need to be more prescriptive in the sense of defining concrete requirements for Data Management and Sharing, for instance regarding where, when, and in which format to share. Especially in Europe, funding agencies often refer to the FAIR criteria [50].

*“For example*, *there needs to be a bigger push to adopt*, *say*, *repositories where such repositories exist*. *And one should perhaps also insist that if data are shared*, *they need to be shared in a format that is open and reusable*. *You can’t just satisfy expectations by sharing data*, *let’s say in a pdf format […] the expectation has to be that data that can be shared in a digital format is shared in a manner that is accessible*. *I mean*, *the FAIR data principles*, *they need to be enshrined in some form in the new policy*. *[…] Things like that […] should probably change*, *that’s where the policy is outdated”* **(Interview 13)**

In particular, funders of projects from different research areas reported that they have to be very careful and considerate in their requirements. They perceive varying degrees of standardisation and experience with data management and sharing within different disciplines. As the task of setting standards and best practices is left to the respective scientific community, these funders are reluctant to specify their requirements and expectations. For example, they do not always explain which data must be shared or what reasons are accepted if requirements are not met. While funders for omics research have a long history of data sharing, funders with a broader spectrum of research funding need to consider different stages of development regarding data sharing practices in different disciplines.

*“[…] the difficulty that we have with the former programmes and what we do as [funding agency]*, *to develop policies*, *is that we are not thematic and […] when it comes to data we have to think about medical research*, *social sciences research*, *earth observation*… *all that means different data*, *different types of practice in the communities*.*”* **(Interview 24)**

Furthermore, some funders reported the need to be rather gentle when implementing their data sharing policy given the lack of a sharing culture in the research community.

*“However*, *we felt that it was too early to have strict recommendations*. *It should be a guideline and like a gentle push*. *But in the 2017 update*, *we felt that it should be*… *the push should be a little harder*. *We should really let them know*, *let the research community know that this is not something that we wish for*, *but something that we actually demand or require*. *[…] However*, *the culture of sharing data wasn’t there and still isn’t in many of the research areas*. *But we’re getting there*.*”* **(Interview 08)**

Lastly, some funders identify a lack of comprehensive alignment, harmonisation, and standardisation of policies with different stakeholders.

*“So*, *we actually saw that because every institution got their own DMP template that they demanded from every researcher […] but we also asked [the researchers] and [other funding agency] did as well*. *So*, *for some research projects that were funded by us*, *they needed to deliver two DMPs and that’s something that we thought »Well*, *that’s not a good thing«*.*”* **(Interview 21)**

### Potential solutions proposed by funders

Several funders hinted at possible or already implemented solutions for some of the aforementioned challenges. As to the problem of a lack of clarity in policies and requirements, some funders emphasised the importance of guidelines and detailed explications of requirements.

*“[…] people need to know what is expected of them*, *because they don’t think about this all the time*. *[…] And so I think if you make your expectations very clear*, *that takes care of a lot of the problems that people have*. *[…] So*, *for example*, *at [funding agency] we have a strong preference that people that are going to be sharing their data are putting it into a data repository […] If they don’t know that we prefer that*, *their plan will not be as good*. *[…] this list of desirable characteristics that I mentioned earlier*, *for data repositories*, *we’re hoping that that can be used as a guide for these people*.*”* **(Interview 39)**

One funder suggests handling diverging discipline standards for research data by providing overviews of existing guidelines. In doing so, funders can inform funded researchers while also raising their own awareness of different research data standards.

*„[Funding agency] tries to provide a certain overview*, *a kind of guideline or recommendation*. *Not like explaining it on a case-by-cases basis*, *but also taking a look at which disciplines already have… so are there any papers or organisations in clinical research trying to work on guidelines*, *for example*.*”* (**Interview 06**)

Some funding agencies emphasised the importance of coordination in national and international networks for Open Science, and drawing on the expertise of such networks is deemed as critical for improving and harmonising data sharing requirements and expectations.

*“There was this ambition to make it easier for researchers and make it less confusing*, *so within [funder network]*, *[funding agency] took the initiative to start a group to look at alignment of [research data management]*. *[funder network] developed a guide for data management plans and that’s why […] we revised our policy*, *because our data management plan is now in accordance with these requirements and guidelines […]*. *So indeed*, *as an organisation*, *we are very well connected and very involved in international efforts around open science in general*, *not just data management*.*”* **(Interview 17)**

### Challenge II: Monitoring of compliance with data sharing policies

Funding agencies increasingly ask funded researchers to hand in some form of Data Management Plan at the early stages of funded projects [Footnote: As with data sharing policies, there are different names for Data Management Plans. Some funders emphasise data sharing more clearly and use the term “Data Sharing Plan”. Others take a broader approach and use terms such as „Data Access and Management Plan”, “Data Management and Sharing Plan”, or “Outputs management plan”. Taking into account these different wordings and conceptual emphases, we group them under the term Data Management Plan or DMP.]. DMPs are supposed to help researchers detail and plan their process of managing research data and making it accessible. For funding agencies, it offers a potential baseline for monitoring, since researchers’ compliance with DMPs can be checked over the course of and at the end of a funded project. However, our interviews show that there usually is no systematic review of (compliance with) DMPs at the middle stages or at the end of funded projects. This suggests a frequent lack of monitoring of researchers’ actual compliance with funders’ requirements.

*“[…] we require them to hand in the DMPs*. *However*, *we don’t follow it up and we don’t do any check on the quality of them*…*”* **(Interview 36)**

Consequentially, agencies often cannot ensure that funded researchers actually comply with their data sharing policies. Potential violations and failures to share data can therefore go unnoticed.

*“The projects cannot start without a data management plan*, *but that is more or less where it ends*. *[…] We don’t check at the end what happened […] for open access we actually have an idea [*…*] I think it is 70% […] of the papers that are published and funded by [funding agency] are made available for open access*. *But we have no idea if people are sharing their data*.*”* **(Interview 44)**

Furthermore, the lack of monitoring may discourage exemplary compliance if researchers perceive funders to be indifferent towards their own efforts.

*“I mean*, *policies are influential*, *but if you don’t enforce or at least monitor them*, *it can also discourage researchers*, *because some of them may make an extra effort to comply*, *but then they see that nobody cares… that might lead to discouragement*. *[…] we want to see data being made available and be available for reuse and that is the challenge*. *We don’t know if that is happening and we don’t monitor it*.*”* **(Interview 02)**

Monitoring compliance can provide information on data management and sharing in funded projects. Conversely, a lack of such information can make it difficult to identify potential problems within the process of research data management and sharing as well as evaluate the effects of the policy. For example, one interviewee pointed to difficulties in determining whether shared data meets certain standards and if it is actually being reused.

*“… part of our issue was that we just had a complete lack of information about that*, *quite frankly*. *We knew that there was an expectation*, *but not really a great deal of information about what the research teams were putting together*… *You know*, *we ask some basic questions in our monitoring*, *so I’m not saying we had no information at all*, *but we didn’t have any depth of information to support that*.*”* **(Interview 15)**

*“Is the data uploaded fine*, *is it FAIR*, *does it respect the standards*, *are we sure that it’s […] really reusable*? *Is it going to be reused*, *has it been reused*? *If all that we are doing to make the data public and open*, *is it just*… *are we doing this just for theory*? *[…] And we had some studies or expert groups*, *so we don’t really have a clear answer*, *it’s very difficult to identify*.*”* **(Interview 43)**

The reason for this deficit is usually perceived to be the same: monitoring requires extensive personal and financial resources, and funders often lack capacities for necessary checks. Any efforts to automate these processes and save resources are often still in their early stages.

*“But there are too many final reports to go through all of them*. *At the moment*, *at least*, *we do not have the resources*, *but we’re trying to make it more automatic starting from next year onwards*, *to get at least the figures and numbers*, *and also maybe the kind of compliance as well*, *if it’s possible*.*”* **(Interview 05)**

Some funders are quite aware of this significant gap between the theory of potential tools for monitoring compliance and putting these tools into practice.

*“We have the tools in place for compliance*, *but I’m not sure we have the execution in place*.*”* **(Interview 29)**

In addition to the aforementioned problems, a number of funders perceives the complicated “nature” of research data to be another major obstacle for monitoring. Ensuring compliance with data sharing policies is perceived to be more complex and resource-intensive than, for instance, ensuring compliance with open access to publications policies.

*“… open access compliance is a lot more [*..*] black and white and the researchers have got much less of an excuse*, *because I’d say we make funds available very readily*, *to make articles open access […] Data is more complicated and it’s much more case by case and obviously the approach varies from project to project and the challenges as well*.*”* **(Interview 10)**

#### Potential solutions proposed by funders

Our interviewees frequently implied the solution of adding more resources to their monitoring capacities, which is demanding in a different way. One funder with significant financial and personnel resources drew a direct connection between its level of resources and its monitoring capacities but also highlighted its interest in alternative, less expensive approaches.

*“I think in general*, *our compliance is probably pretty good if nothing else*, *because we […] have a bit of a labour force to try and help enforce the policy […] But this comes down to what I think is a very important issue*, *which is the issue of resources*. *[…] I could easily imagine a scenario where the policy’s expectations may be difficult to uphold without the level of funding that we might have*. *Probably you could do it in other ways*, *too*. *Certainly*, *the approach that we take is not the only way to do it and I think we’re seeing more and more interest in alternative approaches*.*”* **(Interview 33)**

However, such alternative approaches remain largely unspecified. One potential solution attempted by a few funders is to use digital submission tools such as Researchfish [51] to support the monitoring process, but this does not seem to be common practice. Another approach reported by a number of funders is to trust in the scientific integrity of funded researchers and their willingness to comply with the policies.

*“It has a few restrictions*, *but we very much trust that the people will do what the government asks them to do*, *which sometimes works and sometimes does not*.*”* **(Interview 01)**

### Challenge III: Sanctions for non-compliance with data sharing policies

Our interviews show that funding agencies have several issues with enacting sanctions for non-compliance. This includes questions such as what kind of misconduct can be sanctioned, what sanctions can be applied, and when to impose sanction. Few funders actually sanction non-compliance, and even for them sanctions tend to be considered theoretical due to the lack of concrete enforcement mechanisms.

*“[…] the DMP is going to be mandatory*, *so the sanction that we could have is that if there’s no DMP… this is a research project and there’s no DMP uploaded after six months*… *so in theory*, *there could be a sanction*. *But I think before that the project officer would call the project manager and say »hey*, *can you upload your DMP*, *you’re late« or whatever*. *I mean*, *we don’t have a data police*, *an open access police*.*”* **(Interview 11)**

*“… our lawyers typically tend to be quite [gentle] and also not think of very harsh sanctions*. *Those are just kind of later on checks that we do at the final reports*, *but not exactly […] limitations on whether you can apply for future funding or similar sanctions*. *That’s not possible at [funding agency]*, *at least not at the moment*. *So*, *just maybe reminders and asking for new DMPs*, *if it is not acceptable*.*”* **(Interview 05)**

These passages also reflect a pattern indicated in our data: the interviewed agencies show a strong reluctance towards applying sanctions in cases of non-compliance. When asked about sanctioning, almost all interviewees preferred providing incentives over enforcing sanctions. Usually they would opt for diplomacy before seriously considering sanctions.

*“I will also say that oftentimes whenever there is a problem*, *[…] for example data can’t be shared or whatever*, *cutting off the funding is not going to make it be shared*. *[…] So*, *I think the sticks are particularly shaped*. *They don’t necessarily compel the behaviour that you need*, *[…] it’s much more about diplomacy than anything else and sort of being creative and coming up with solutions that actually work for people*.*”* **(Interview 19)**

*“[…] as long as the grantees somehow say »Oh*, *I didn’t know« and »Sorry« and »Next time better« and whatever*, *you won’t apply such sanctions*. *So*, *no*. *That is inconceivable*. *And that would really be a bit inappropriate*. *As long as there really is any goodwill… and if you persuade people long enough*, *a good intent is always there*.*”* **(Interview 04)**

Some funding agencies consider incorporating sanctions into their policies, although some may have reservations as they are deemed less efficient than incentives when it comes to incentivizing data sharing. Only a few interviewees acknowledged minor benefits of sanctions.

*“The carrot is always going to make more of an impact than the stick in the long run*. *In the short run*, *the stick has more impact*, *but in the long run the carrot has more impact because it’s a mentality shift*.*”* **(Interview 07)**

Apart from general preference for incentives, the complexity of data sharing causes some funders to be very careful when it comes to potentially premature sanctions. In comparison to open access to publications, there are more technical, ethical, and legal difficulties to consider, as well as the aforementioned differences between discipline standards.

*“So*, *at the moment we don’t have any specific sanctions for when data isn’t shared in line with our policy*. *I think*, *it’s something that we’ll continue to review over time*. *[…] it’s likely that we’ll […] think about more formal sanctions over time*, *but as I said*, *we don’t have them at this point and probably still think it’s premature*. *So*, *for open access to publications it’s a slightly different situation*, *there is a lot more black and white […] As I said*, *it’s harder to introduce those things for data”* **(Interview 35)**

A final issue with applying sanctions for non-compliance lies in the question of when to apply them. On the one hand, some interviewees conceded that financial sanctions for non-compliance reach a limit as soon as funded projects end and the last tranche of funding has been paid.

#### Potential solutions proposed by funders

On the other hand, funding agencies can still sanction researchers refusing to share data by taking these actions into account when evaluating researchers’ applications for future grants. While some interviewees consider it as an instrument to sanction non-compliance, others highlight it as an incentive for sharing data.

*“… we really can’t hold on to moneys after the award ends*, *so all we can do in terms of sanctions if someone doesn’t publish subsequently and we haven’t got any money to withhold*, *all we can do is that in a future application*, *[…] if it was awarded*, *they wouldn’t actually get those funds until they published the previous trial*.*”* **(Interview 32)**

As some interviewees noted, sanctions require funders’ demands regarding data sharing to be clearly articulated. Without this, funded researchers may not know how to comply with the policies and what consequences might follow from non-compliance. A few funders consider withholding parts of the funding as an appropriate approach when researchers do not meet clearly set requirements.

*“So*, *for the length of time that they’re awarded*, *if they’re not complying with the timeline that they submitted to [funding agency] on when they’re going to share*, *[funding agency] can withhold the funds for that year until they share what they said they were going to share*. *So*, *this is all a bit of a stick there”* **(Interview 19)**

As a general pattern throughout our interviews, funding agencies appeared to be hesitant, if not reluctant about sanctions. Instead, they repeatedly expressed hope for a cultural change to happen.

*“I think it’s the opinion of [head of funding agency] that we shouldn’t have to be*… *his hope and his aim is that in a couple of years*, *this will just be the norm anyway and then there won’t really be any need for sanctions*.*”***(Interview 40)**

### Challenge IV: Incentives for data sharing

Among funders, there seems to be a broad agreement on the benefits of carrots over sticks. However, most of our interviewees concurred that there is a serious lack of incentives for data sharing. Some of them even noted that the competitive state of the scientific system may even impose contrary incentives for data sharing.

*“[…] it means that when people share data […] it’s basically forcing them to work against the incentive structure that we have now*, *where the incentive*, *because of the publication*, *is to withhold your data and to get as many publications out of it as you can*, *or to force people to collaborate with you in order to get access to it*, *so you can be a co-author on those publications*. *And we just don’t have the incentive structure in place unfortunately as a community*, *not just [funding agency]*, *to really reward people for when their data are made available and used frequently*.*”* **(Interview 33)**

Some funders see the need to come up with better incentives in order to shift the balance towards data sharing. While they appear to be aware of the general lack of incentives (and the existence of contrary incentives) for data sharing, concrete solutions to this issue are not trivial. Despite several efforts to address the perceived lack of incentives, many of them still seem to be in the planning stage and remain rather vague. Therefore, funders repeatedly struggled to give concrete answers as to how exactly they want to implement better incentives.

*“*… *the hard problem is how do you incentivise data sharing*? *That’s the nut to crack first*, *or at least that’s the nut that we want to crack*…*”* **(Interview 27)**

Several funders recognise that they can or even need to do more in terms of providing incentives. They identify the problem that researchers receive too little reward and recognition for data sharing, and see (often unspecified) room for improvement for their own efforts.

*“I think we do need to look much harder at the [incentives]*. *We give very little credit*, *I think*, *at the moment for data sharing*. *So*, *however much we go on about it*, *we don’t really*… *we don’t look at it in applications*, *to see whether data has been shared or value it as*, *you know*, *a data publication*, *in the same way as a publication*, *a research publication […] So*, *we do need to be more creative in that area*.*”* **(Interview 32)**

Another challenge for incentivising data sharing lies within the organisational structure of funding agencies themselves. Some interviewees pointed to a certain reluctance towards open science within their own agencies, for example at the level of grant review committees. As a consequence, funders’ efforts to incentivise data sharing may be hampered.

*“[…] the committees themselves can be quite conservative*. *[…] it’s still a difficult topic within the organisation to reward researchers for open science*. *A lot of people are still a bit scared of that*.*”* **(Interview 44)**

*“[…] I think [funding agency’s] very conservative approach to this is misinformed*. *I think that the people at the very top*, *like those centre directors*, *have been around for a really long time and they’ve got this culture of competition*, *kind of as part of their scientific being*. *[…] And we see*, *of course*, *the whole ecosystem of science and scholarship changing*, *you know*, *to where people are urging that their data […] become an equal product of research to a publication and so forth*. *And I think this very conservative approach that [funding agency] has taken with this policy*, *and I think it’s good*, *but I think it’s conservative*, *it could be a lot less conservative and I think we could advance open science a lot faster […] than they think*.” **(Interview 12)**

#### Potential solutions proposed by funders

The interviewees emphasised that the way researchers are evaluated has to change in a way that considers data sharing as a valuable contribution to science. According to them, funders can contribute to this by pushing their grant evaluation metrics in a direction that more strongly considers data sharing and other contributions to open science as valuable scientific outputs. This approach is in line with the goals of several initiatives and declarations [52, 53], which were also mentioned by the interviewees.

*“And I think that that is something that we need to work towards*, *I think it’s something that greater use of persistent identifiers and tracking metrics of data use could certainly help with*. *I think funders*, *not just [funding agency]*, *but others and institutions like universities*, *need to pay attention to these metrics and need to start considering them in things like tenure decisions or hiring decisions*.*”***(Interview 16)**

A number of funders considers a more widespread application of tools like persistent identifiers for authors and datasets, in order to improve recognition for scientists sharing their data. Other potential incentives mentioned in the interviews include, for example, public acknowledgment of data sharing by researchers on the homepage of funding agencies or nudges.

*“We have to think of little nudges and little things that say […] »Hey look*, *that data set that you put up there*, *5000 people have already downloaded it and there’s this other paper*, *that’s using this data that you published*. *That’s amazing*! *Let’s highlight that*, *[…] some researcher somewhere else is using our data and that’s awesome*.*«”* **(Interview 38)**

In order to shift the balance between the costs and the benefits researchers see in data sharing in favour of the latter, funders report the need to better highlight individual benefits for researchers.

*“Now I personally think there’s a good chance that scientists actually benefit in their own careers from data sharing*, *because the earlier they share*, *the more they share*, *the more other people can use that and that actually reflects back positively on them*. *So*, *I think this notion of »oh*, *I get scooped if I share« is*… *I mean*, *it’s out there and it’s very powerful*, *but I think it’s ultimately a red herring*. *[…] maybe as a community*, *we just have to do a better job at showcasing that sharing benefits not just society and science in general*, *but it benefits the individual scientist who shares*. *I think that message needs to be gotten out better*. *[…] If they accept »oh*, *it’s better for me to share because I benefit«*, *that barrier would be gone*.*”* **(Interview 46)**

Finally, some funding agencies hope for cultural change as an ongoing process in which data sharing becomes both an integral part of good scientific practice and an intrinsic motivation for researchers.

*“I’d say a lot of the challenges are still very much there and the one I would definitely highlight is the question around incentivising and […] data sharing becoming [*…*] embedded as sort of a standard part of research practice*.*”* **(Interview 25)**

### Challenge V: Support and guidance for data sharing

Researchers face a number of hurdles like additional workload, legal obligations and ethical responsibilities when managing and sharing research data. Several interviewees were aware of these problems and expressed the need to make data sharing “as easy as possible” for funded scientists.

*“Actually*, *this was quite common*, *people just wanting template text that they could put on the DMPs because they found it kind of bureaucratic and a pain*.*”* **(Interview 17)**

*“I think the issue is that open access or open science require more burden of work*. *[…] And any little burden that you add on top of it makes it difficult*. *I think that’s the stress*. *And this is the thing that we as funders and the publishers […]*, *that’s what we need to work on*. *[…] in the end*, *we’re trying to facilitate that good science gets done […] And we need to kind of make things as easy as possible to do that*.*”* **(Interview 07)**

However, our interviews show that “making it easy for researchers” is not all that simple. Funding agencies often try to identify the prevalent barriers and burdens for researchers, but sometimes struggle with a lack of information due to a lack of feedback and monitoring. Funders in smaller countries (with smaller national scientific communities) seem to communicate more directly with funded researchers.

*“[…] we’re currently having an inquiry […]*, *we sent out a bunch of questionnaires*. *We have actually sent them out to find out exactly that*. *How is it going*? *What do you think about it*? *How is it going in your field*? *So*, *I don’t have an answer now*, *but we are actually planning to put that together”* **(Interview 30)**

However, large funders sometimes seem to demonstrate a certain disconnect between their own policy level and funded researchers. Especially for major funding agencies that outsource parts of the management and evaluation of funded projects to external contractors, lacking contact with researchers points to a lack of feedback on actual hurdles.

*“So*, *all of these feedback mechanisms et cetera are not exactly what you would like them to be […] I think that has a lot to do with this separation*, *that on the one hand there’s the policy at [other funding agency]*, *and on the other hand is the project management*, *in the sense of grant management*, *evaluation management and so on and so forth*, *everything is outsourced*. *With evidence-based decision making*, *it’s not necessarily always so easy*.*”* **(Interview 23)**

Based on our interviews, many funding agencies do not provide concrete support, such as technical or legal assistance. Instead, they perceive their primary role in fostering data sharing by providing funding, raising awareness and offering guidance. While this can also be considered a solution approach to some extent, there still seems to be a lack of awareness: Some interviewees brought up funded researchers not being acquainted with data management or unaware of supporting means. For example, a number of agencies are willing to provide projects with additional funds for data management, but researchers are not always aware of this.

*“Researchers sometimes still lack an overview and […] a certain guidance could be helpful*. *If you say »Okay*, *these are from us*, *for example recommended repositories*, *or these are especially often used by the community«*, *this could also be offered as an aid to the researchers*. *Then there is the whole financial aspect*, *[…] many researchers have not applied for additional funds for data preparation and upload [*…*] simply that you maybe increase awareness a little more*, *that data sharing comes with an additional effort*.*”* **(Interview 31)**

On top of this, some interviewees exposed the problem of researchers also not being informed about the help they can get at their own institutions with regard to data management. Therefore, some funders reported the need to provide orientation and guidance for funded researchers.

*“Sometimes researchers are not aware of the facilities provided by their own institutions because often […] they know what’s available in their communities*, *but not what’s available within the institutions*. *So*, *it serves the purpose of bringing researchers in contact with support staff and make sure they are aware of the facilities and the tools and that they can use for data management*.*”* **(Interview 02)**

Our interviews indicate that funding agencies not only fund researchers, but also invest money in universities, libraries, and repositories to build up capacities and a supportive environment for data management and sharing. However, efforts like these require significant financial resources from funders and research organisations to develop support infrastructure for research data. Funders have different levels of resources at their disposal, which is why supporting capacities are not always sufficient.

*“… there is now a funding programme […] to provide funding to the universities to further build up their researcher support offices and data stewardship*, *but that capacity is not in desired strength yet*. *So*, *that is a challenge*, *but everybody is convinced that this is the way to go and also the universities are now convinced that they should invest in data stewardship and open science support offices*.*”* **(Interview 03)**

#### Potential solutions proposed by funders

When it comes to supporting data management and sharing, several funders perceive most practical expertise and supporting capacity at research institutions themselves, for example at universities. Therefore, funders delegate the task of supporting funded researchers to universities.

*“I think we can say that these requirements are […] a driving force for universities to expand their expertise*, *because we as [funding agency] do not have*, *or at least our colleagues do not have*, *the full expertise on data management*, *and that’s why we give the responsibility back to the universities*. *And this change in the policy actually led to an increase in support offices in universities*. *So now*, *researchers have way more expertise at their institutions and where they can get help on these issues with FAIR data*.*”* **(Interview 03)**

This points to a division of labour: funding agencies provide general guidance for researchers and support research infrastructures financially, which in turn support funded researchers with their legal and technical expertise, for example when it comes to writing DMPs. Other than this, the supporting efforts by funding agencies reported in our interviews were rather heterogenous and did not indicate a one-size-fits-all approach.

### Challenge VI: Limits to the capabilities of funders

One last prevalent challenge from our interviews addresses the general scope of action of funders in the context of data sharing. On the one hand, they repeatedly expressed the desire to help create a fertile data sharing environment and help to induce a cultural change.

*“[…] we have done a lot of work as a funder*, *thinking about […] the broader landscape*, *how we support the policy and provide guidance to researchers*, *but also how we address some of the broader kind of challenges in the environment around data sharing*, *of which there are several*. *There are challenges around developing and supporting the infrastructure required for data sharing*, *challenges around […] incentives and creating the right […] culture for the kind of recognizing and rewards*.*”* **(Interview 10)**

On the other hand, they also perceive a number of challenges concerning their own capability of implementing significant change in a proactive way by themselves. As illustrated above, they perceive data sharing to be very complex, for example due to different community standards.

*“[…] because it’s so complex and because it’s at the forefront of research*, *it’s really hard for an organisation to simply put the foot down and say »This is what we*…*« I mean*, *it’s more about how*… *maybe shepherding a community practice or community standards […] that is what is needed*.*”* **(Interview 13)**

Our interviews point to several limiting factors to funders’ capabilities. First, there are number of competing interests that complicate the practice of data sharing. This includes potential conflicts with companies or publishers, usually regarding commercial and legal aspects around research data (e.g. intellectual property).

*“[…] when we started with research data and openness in our former programme*, *we called it "open research data" […] That freaked out some people*, *especially companies*, *because they get the impression that*, *you know*, *the openness of research data had to be the same as that of publications*.*”* **(Interview 43)**

There are also potential disputes with other stakeholders within the scientific system like funded researchers, but also scientific communities at large. A common cause seems to be that certain measures by funding agencies are not considered appropriate or legitimate and are thus met with resistance. As mentioned in Challenge IV, there are even disagreements within some agencies.

*“And I think the backdrop for this*, *for the expansion of [funder’s policy] and some of its provisions […] it was a debate that went on for something like seven years and didn’t ultimately result in a change because of the amount of pushback*, *I think*, *from the research community and some institutions and concerns about the complexity and the difficulty of doing that*.*”* **(Interview 19)**

One interviewee named the absence of an official governmental strategy on open science as a major obstacle to their capabilities: whereas funders can only influence funded researchers, governments can evoke a more comprehensive strategy and therefore support the efforts of funders.

*“*S*o*, *what we can do is we can put demands on the people that get funds from us*, *but we cannot put demands on researchers or universities that do not get funds from us*. *My point is that the government can do that because they […] give the funding to universities*, *but we only give funding to the researchers and obviously not everybody*. *[…] We certainly have been proposing to the government what the strategy should be*. *Our government still does not have a strategy for open access to research data and it is the government that needs to put that strategy in place*.” **(Interview 45)**

Data sharing comes with a number of restrictions, some of which pose legal or ethical limitations to the efforts and requirements of funding agencies. One major example is protecting the privacy of study subjects for reasons of confidentiality. In this case, funded researchers may have legitimate reasons not to share their data.

*"But we just have this awareness*, *which we also express in the policy*, *[…] that there can be ethical or legal problems*, *for example when you make data available*. *And we just point that out*, *that if there are [such problems]*, *that you can then also deviate from this policy of data sharing*.*"* **(Interview 09)**

*“And we’re not compelling people to share [data]*, *we are compelling them to tell us how they are going to manage [data] and we expect them to maximise their sharing*, *but we provide many reasons why you might not be able to share to the fullest extent possible”* **(Interview 16)**

Finally, some funding agencies seem to struggle with different aspects surrounding research data management and sharing. One interviewee indicated that funding agencies might even need support and guidance themselves.

*“[…] we had also four years to train our own colleagues in open access and research data management and what is our policy […]*. *So*, *we still had to do that and we still have to*. *So*, *at some point it was also too much for two or three people and we were just like »we need professionals also to help us«”* **(Interview 11)**

#### Potential solutions proposed by funders

Several funding agencies take a collaborative approach by pooling expertise through international and national networks. Initiatives like Science Europe [45], Research Data Alliance [54], or cOAlition S [55] allow for shared standards around open science, learning from each other, and building a fertile data sharing environment.

*“But we always try to coordinate ourselves very well internationally […] for two years now we have also been a member of [network]*, *this other association of funding agencies*. *[*…*] This is also a group of stakeholders with whom we network very closely*, *if only because we are of course now also adapting the policy and then discussing together in this group of funding agencies how we can take steps together*, *adapt policy*, *change policy in order to achieve the common goal*.*”* **(Interview 20)**

Exchanging know-how and information seems to be crucial for funding agencies, since they can learn from and coordinate with other organisations. This not only has practical implications but, as one interviewee pointed out, is also in the spirit of open science.

*“I mean*, *we are not innovating*, *in a way we are sort of*, *actually in the spirit of open science*, *»reusing« [other funding agency’s] ideas*, *because we also talked to them*. *It’s also in the spirit of open science not to reinvent the wheel if there is something already out there*.*”* **(Interview 44)**

In addition to cooperating with other funding agencies, there is also the option of engaging with other stakeholders to find common ground in terms of data sharing policies. This includes the collaboration with research organisations, but also with journals and different research communities.

*“[…] so that is a challenge there*, *but generally we find that awardees are very*… *do comply because it’s*… *compliance is not only with the [funding agency]*, *we find a companionship with compliance with journals*. *Because when you go to a journal*, *when you’re ready to publish*, *they expect you to submit your data*.*”* **(Interview 19)**

Furthermore, dedicated governmental strategies and initiatives on Open Science (such as the principles by the Organisation for Economic Co-operation and Development) can lend additional legitimacy to the efforts of funding agencies.

*“[…] the element that has supported the creation of that policy was the OECD principles on sharing data […] from publicly funded research*. *And that I think was part of the reason*, *because being an OECD member*, *[country of the funder] would have signed to these principles and it supported the development of the policy from a political perspective”* **(Interview 22)**

### Overview of challenges

Table 1 presents a summary of our results, laying out the overarching challenges we identified in the interviews, different aspects of these challenges, and the proposed solutions by funders. Note that the solutions presented here refer to the challenge in general and are not directly connected to the aspects next to them.

**Table 1:** Overview of challenges perceived by funding agencies and solution approaches.

Name of the challenge	Aspects of the challenge	Proposed solution
Challenge I: Design of data sharing policies and requirements	◾ Lack of clarity ◾ Consider different discipline standards ◾ Need for shared efforts	◾ Guidelines and detailed explications ◾ Provide overviews on best practices and raise awareness ◾ Engage in funder networks
Challenge II: Monitoring of compliance with data sharing policies	◾ Lack of monitoring ◾ Discourage researchers ◾ Shortage of information ◾ Lack of capacities and resources	◾ Additional resources ◾ Alternative approaches like automated checks ◾ Trust in researchers
Challenge III: Sanctions for non-compliance with data sharing policies	◾ Lack of enforcement mechanisms ◾ Reluctance towards sanctions ◾ Complexity of data sharing ◾ When and how to apply sanctions?	◾ Clear prescriptions and grant conditions ◾ Hold back part of funding ◾ Consider future grants ◾ Hope for cultural change
Challenge IV: Incentives for data sharing	◾ General lack of incentives ◾ Struggle to provide concrete incentives ◾ Too little reward and recognition for data sharing ◾ Conservatism towards data sharing	◾ Change grant evaluation metrics towards data sharing ◾ Better recognition and acknowledgment ◾ Hope for cultural change
Challenge V: Support and guidance for data sharing	◾ Fund data sharing infrastructure ◾ Lack of information due to lack of feedback and monitoring ◾ Disconnect between policy level and researchers ◾ Funded researchers lack awareness of funders’ and institutional support	◾ Make it easier for funded researchers ◾ Hands-on support by research organisations ◾ Funders provide general guidance ◾ Funders try to make it “as easy as possible”
Challenge VI: Limits to the capabilities of funders	◾ Different stakeholders and community standards ◾ Conflicts with other stakeholders and within funding agencies ◾ Dependence on other stakeholders ◾ Ethical and legal boundaries	◾ Shared efforts by funder networks ◾ Learn from other agencies ◾ Collaboration with journals and research organisations ◾ Governmental strategy on research data

## 4 Discussion

We conducted 16 guideline-based expert interviews with representatives of international funding agencies in order to investigate their perspective on their own data sharing policies. Our interview data revealed different problems faced by funding agencies concerning their data sharing policies, which we categorised into six broader challenges. The goal of this article was to illustrate these challenges and, to a lesser extent, to highlight potential solutions mentioned in the interviews. Accordingly, we will focus on the challenges for funders in the following discussion, but also briefly touch on what we perceive as plausible solutions reported by the interviewees. We discuss our findings both in light of other literature as well as the premise that sharing research data for replication and reuse benefits the scientific community and the public. This view is shared by many stakeholders in the scientific community [7–10].

Our interviews indicate that there appears to be a challenge for funding agencies to clearly articulate what they expect funded researchers to do in terms of data sharing and data management. This need for more clarification in data sharing policies is reported in several of our interviews as well as other research [1, 39, 40]. Clear requirements appear even more crucial in light of other studies identifying funder policies as important guidelines for many researchers [11, 36–38]. Our interviews suggest that part of the problem is addressing different community standards because different disciplines are at different stages of development and there is no one-size-fits-all approach, which is reported in other studies as well [12, 56]. Furthermore, our data confirms observations that funding agencies face difficulties concerning the implementation of policies and are themselves in different stages of development [30]. The most concrete and plausible approach to these challenges indicated by the experts referred to coordination with and learning from other funders to align their policies, exchange information, and establish best practices and shared standards.

Funders report that they hardly check whether and to what extent grantees comply with data sharing policies, which indicates a wide lack of monitoring of compliance of researchers’ behaviour regarding data sharing. This is a somewhat surprising insight from our interviews and–with some exceptions [30, 36]–it remains underexplored. A lack of monitoring is noteworthy, since it poses at least three risks: First, it may send questionable signals and discourage researchers who are motivated to comply with the rules outlined by data sharing policies. Second, a lack of monitoring opens the door for rule violations and might lead to unfairness towards complying funded researchers. Third, a lack of monitoring causes a lack of information about data sharing by funded projects, which in turn makes it more difficult for funders to evaluate and adapt their policies. The frequently suggested solution of investing more resources into monitoring raises the more general question of resource allocation for funders. Other approaches suggested in our interviews do not seem to be established, for example the use of Researchfish, or are rather passive and questionable, for example simply trusting researchers.

Most interviewees report that their organisations do not provide sanctions or describe them as more of a hypothetical option. Furthermore, the reported lack of monitoring undermines any implementation of sanctions. Funding agencies perceive several problems with sanctions, some of which are issues out of principle, such as a general preference for incentives over sanctions. Others raise questions about which sanctions to use or what kind of behaviour to sanction. Funders’ broad reluctance towards sanctions is noteworthy in light of studies on the success of journals’ data sharing policies, which appears to be connected to their ability to enforce them [57–59]. Some studies also suggest that enforcement mechanisms can be functional for implementing data sharing policies of funders [39, 60] and that many researchers deem sanctions legitimate if grantees do not comply with data sharing policies [37]. As for funding agencies, there are potential sanctions at hand. Some interviewees suggested exclusion from future funding, for example. However, the implementation of any potential sanction would require a solution to the two aforementioned challenges concerning clearly articulated and comprehensible grant conditions as well as monitoring researchers’ compliance with data sharing policies.

In light of their expressed preference for incentives over sanctions, it may come as a surprise that funders admit to having a lack of incentives in place. They are aware of the problem that researchers receive too little reward and recognition for data sharing. The underdevelopment of an appropriate system of incentives for data sharing seems to be a particular problem considering the evidence that incentives like proper credit and career benefits seem to be a key motivator for researchers to share their data [1, 19, 38, 61, 62]. The lack of effective incentives is also reported by other observations [3, 36]. Approaches that were repeatedly mentioned in our interviews include greater visibility for researchers who properly manage and share their data or changes to evaluation metrics altogether by considering data sharing in grant proposal evaluations, final reports, and researcher evaluations. Efforts like this are also suggested in other work [63].

Our interviews indicate that funders support data sharing in various ways, like providing guidance to researchers, additional financial means for data management, and funding for local support infrastructures. The latter also implies that funders expect the host institutions of funded researchers to offer support on technical, legal or ethical issues pertaining to data management and sharing, for example by local data stewards. On the one hand, this outsourcing of support comes with the risk of a decreased communication between funders and their grantees. There are numerous problems around data sharing for researchers reported in literature [14, 25, 40], and it is important that funder policies do not lose sight of these issues. On the other hand, this division of responsibilities between funders and research organisations seems to be a reasonable approach, since the local support may be closer to the specific needs of researchers. Funders can live up to their responsibility by making clear what kind of support they can offer to funded researchers, such as organising information events or workshops for funded projects, and providing detailed information on their homepage.

According to our findings, there are two types of limitations for funders’ efforts to foster data sharing: First, there are external limits to the capabilities of funders, such as disagreements with other stakeholders and scientific communities, the dependence on research organisations for support or the general complexity of the issues at stake. Second, there are internal limitations, such as a reluctance or even resistance towards open science within funding agencies, a lack of practical expertise, and a lack of resources. Some proposed solutions reported in our interviews, such as the collaboration with other stakeholders, the joint development of shared standards, and learning from other funders are also suggested in other studies [30, 36, 64]. Some of our interviewees suggested that dedicated governmental initiatives can also help raise awareness for the importance of data sharing and provide data sharing policies with political legitimacy.

These observations and reflections lead us to some conclusive remarks. Based on the assumption that data sharing benefits science, the scientific community, and the public, the overall picture of funders’ data sharing policies emerging from our interviews shows a lot of room for improvement. There are several important structural and interrelated challenges: A lack of clarity of requirements, widespread absence of compliance monitoring, lack of evidence about data sharing behaviour, and insufficient incentives. These shortcomings threaten to undermine the general efficacy of the entire system of funders’ data sharing policies. In our interviews, many funders were aware of these deficits and named strategies and solutions to address them. Despite this, we are sceptical towards an approach mentioned by some funders, which is to wait for a cultural change towards data sharing. There is no guarantee that such a cultural change will emerge on its own. Certainly, funders alone cannot enforce a cultural change, and multiple issues reported in our interviews cannot be solved by a single stakeholder, let alone a single funding agency. However, funders can be leaders and trendsetters in this development, as data sharing policies are important instruments for guiding research data culture and practice.

### Limitations

Building on the methodology of qualitative empirical research, there are several limitations that apply to the results of this study. First, the expert interview method assumes “experts” to be somewhat representative of their respective fields. Since the funding agency employees interviewed for this study mostly belong to large or innovative agencies, they represent the state of the art to some degree. However, there are funding agencies that are less developed, face other issues or even lack data sharing policies overall. Second, the scope of the study is limited by the number of interviews conducted. Although they are substantial sources of (qualitative) data, we do not claim to offer an exhaustive perspective on the challenges faced by funders in general but draw conclusions from the issues that came up in our interviews. Third, and although we are using interview guidelines, there are variations in the questions and the contexts of the interview. Therefore, the interview utterances are not easily comparable. It is therefore methodologically problematic to give clear quantitative statements such as, for example, “7 out of 16 interviewees said X”. Fourth, the perceptions and experiences confidentially shared by the interviewees may differ from the official position of their organisation, which was explicitly stated by some interviewees. Finally, some of our interviews took place more than a year ago, and the respective data sharing policies may have changed in the meantime.

## 5 Conclusion

It is a widespread notion that research funding agencies can play a key role in promoting data sharing by implementing data sharing policies. However, there is limited evidence on how funding agencies themselves perceive opportunities, limitations, and their own role in promoting data sharing. The goal of this article was to contribute to filling this gap. Based on the data collected in 16 interviews with open science contacts at international funding agencies, we identified six major and interrelated challenges: The design of clear policies (I), monitoring of compliance with these policies (II), sanctions for non-compliance (III), providing incentives (IV), offering support (V) and the limits of funders’ agency (VI). Our interviews point to critical flaws in funders’ data sharing policies such as a lack of clarity, a lack of monitoring of funded researchers’ data sharing behaviour, and a lack of incentives. A number of agencies are aware of potential solution approaches but struggle with the practical implementation of these measures. Funders cannot solve these challenges all by themselves, but they can play a more active role and lead joint efforts towards a culture of data sharing.

## Supporting information

S1 Appendix Interview guideline. (DOCX)

S2 Appendix Transcription and coding guideline. (DOCX)

S3 Appendix Category system. (DOCX)
