# Supplementary material for: Neither carrots nor sticks? Challenges surrounding data sharing from the perspective of research funding agencies—A qualitative expert interview study
Source: PLoS One. 2022 Sep 7;17(9):e0273259. doi: 10.1371/journal.pone.0273259 (PMC9451069; doi:10.1371/journal.pone.0273259)
Supplement: S1 Appendix — (DOCX) [file pone.0273259.s001.docx]

**Introduction to the expert interview**

Thank you so much for taking the time. **Prior to the interview, you received some information** on our project and the context of the interviews, as well as a declaration of consent. I would now like to start by summarizing the most important aspects and requesting your consent.

- Our research project is called „Carrots and Sticks? Data Sharing Policies for (German) Public Research Funders. Ethical, Legal, Social and Behavioral Aspects (Datablic)“. It is **funded by the German Ministry of Education and Research** (BMBF).
- We investigate how public research funding agencies could and should design their funding policies, particularly their data sharing policies, in order to ensure that funded projects share their research data with the scientific community. **Our overarching research goal is to develop scientific recommendations for the German funding agencies**.
- We conduct expert interviews with representatives of organisations we found progressive in terms of their research funding policies in terms of data sharing. We are particularly interested in your personal experiences and perceptions in this area and want to gain insight into state-of-the-art data sharing practice. **We would like to record and transcribe the interview, in order to perform a comparative content analysis later on**.
- All audio and text material based on the interview will be **treated confidentially**. It will be **anonymised**, i.e. all personal references that can be used to draw conclusions about you will be deleted, the file itself will be **pseudonymised**. This also applies if we cite certain parts of the interview as part of a publication. All associates of the project are bound by confidentiality. Data won’t be stored in any data cloud, files will be password-protected at all times.
- Please note that a **revocation of your consent is possible at any time**, as stated in the information document. You can chose to not answer questions, to pause or to end the interview at any time, without providing any reasons and without any negative consequences for yourself. **You have the right to review the interview transcript.**
- **Do you have any further questions, for example about the project or the interview procedure?**
- **I would like activate the audio now, in order to request your verbal consent. Do you agree to this?**
- **Obtaining oral consent (based on DATABLIC consent form):** “I confirm that I have read the information form and have been informed about the purpose and procedure of the study. I had the opportunity to pose questions. All of my questions were answered to my satisfaction. I consent to voluntarily participate in the study and I was granted enough time for my decision to do so. I received a copy of the information form and the declaration of consent in advance, and I took note of them. I hereby agree with the procedure detailed in the Datablic Information Sheet and explained by Mr Michael Anger. I consent to the recording of the interview and the use of the anonymised data gained in the interview for the sole scientific purpose of the datablic project. **Would you please both state your name and say whether you consent with these terms?**
- The interview consists of four different, broader topics that include several questions. I would like to ask you to talk about everything that seems relevant to you, take as much time as you need for your answers. As told in advance, the interview takes approximately 45 minutes.

| **Topic 1: Development of and participation in funding policies on data sharing (10 Minutes)** |  |
| --- | --- |
| 1. Our main research interest is the topic of open data, with a focus on the process of data sharing. Since you are very involved in the topic of research data (management/sharing/policies) within <organisation>, could elaborate a bit about the current status quo of your organisation’s policies on research data (management/sharing)? 2. With regard to the development of these policies: Do you know if there was any guidance from or participation of …  - Other stakeholders like the government, the scientific community, other funding agencies, … - How did this participation look like? What results came from it?  1. Could you elaborate, if there were specific legal (constitutional, data protection law) and/or ethical problems considered in the development of your policies?  - Did you face any ethical problems? Any particular legal obstacles, laws/regulations that you had to consider? - (privacy, intellectual property?) What were the results of these considerations?  1. Data sharing is a complex process that includes several stakeholders [data donors, data producers, secondary users, scientific communities, journals, the public]. How do you consider the interests and needs of different stakeholders in your data sharing policies? |  |
| **Topic 2. Experiences with data sharing policies (benefits, boundaries and hurdles) (10 Minutes)** |  |
| 1. Let’s talk about your own perspective on and experience with your organisation’s data sharing policies.  - Which impacts do you see? [What are potential benefits?]  1. Which hurdles/problems/conflicts do you perceive when it comes to data sharing in general?  - Which problems do you perceive related to your policies? - How are these addressed and solved by your funding policies? [e.g. technical/legal/political/ethical/motivational/economic barriers?]  1. How do funded researchers handle mandatory data management/sharing (plans) from your point of view?  - What kind of responses/feedback do you receive? - Does the practice seem feasible to you? - Do you offer guidance on best practice?  1. Do you perceive a tension between mandatory funding policies with regard to data sharing and academic freedom of researchers?  - If so, is it considered and weighed in your funding policies?  1. Are you (currently) considering any changes or updates to your funding policies? |  |
| **Topic 3. Stakeholder’s options, rights and duties with regard to data (sharing) (15 Minutes)** | |
| 1. One of the greatest benefits of data sharing is the improved accessibility of research data. What role does your agency play in ensuring data access to secondary users?  - What are the responsibilities/duties of a funding agency in this regard?  1. Who is responsible for the quality and safety of data? 2. How does your funding agency handle intellectual property and copyright claims of funded researchers when it comes to (raw) research data?  - Do you perceive ownership of data as contrary to the idea of data sharing?  1. How do you address the rights and needs of data donors/study participants (confidentiality, privacy) in your funding policies?  - Is personal data treated distinctly/exceptionally? - Do you have any guideline on addressing different types of consent if the research data is personal? [e.g. informed/broad/… consent?] | |
| **Topic 4. Monitoring, incentives and sanctions (10 Minutes)** | |
| 1. From our exploration of data sharing policies, monitoring mechanisms are widely unspecified (or not very specific) by funding agencies.  - How binding/mandatory is the fulfillment of data sharing expectations/requirements in policies? - How do you monitor compliance with data sharing policies? - How do you ensure that research data is uploaded to data repositories by your own funded researchers? - Is there any way to opt-out of your policies? If so, what are legitimate reasons not to share data?  1. What sort of sanctions do you enforce if there are any violations?  - Are these potential sanctions transparent and clearly communicated? - Have any sanctions been enforced yet? - If so, what kind of sanctions? - What were the sanctioned violations?  1. A common perception is that data sharing requires incentives for researchers. How do you incentivise researchers to share their data?  - Do you consider previous Open Science dedications to be part of future evaluations? - How do you support funded researchers to comply with your policies? - Carrots and Sticks - How should they be balanced in your opinion? | |

**Addendum:** For more detailed information about the guideline please contact the corresponding author: [Michael.anger@dkfz-heidelberg.de](mailto:Michael.anger@dkfz-heidelberg.de) / ORCID <https://orcid.org/0000-0002-9328-510X>
