# Supplementary material for: Neither carrots nor sticks? Challenges surrounding data sharing from the perspective of research funding agencies—A qualitative expert interview study
Source: PLoS One. 2022 Sep 7;17(9):e0273259. doi: 10.1371/journal.pone.0273259 (PMC9451069; doi:10.1371/journal.pone.0273259)
Supplement: S2 Appendix — (DOCX) [file pone.0273259.s002.docx]

**Transcription and Coding guideline for qualitative content analysis of DATABLIC expert interviews**

**Preliminary remarks:** The function of this document is to serve as a general guideline for all the interview transcriptions and the coding of data gained by our expert interviews. In doing so, it provides a set of coding rules, which were established by discussions within the social sciences subproject. For a list of all categories, see the attached category system. For other requests, such as access to the complete codebook, please contact the corresponding author: [Michael.anger@dkfz-heidelberg.de](mailto:Michael.anger@dkfz-heidelberg.de) / ORCID <https://orcid.org/0000-0002-9328-510X>

**Transcription guideline**

A. "Filler words" such as “um”, “err”, “uuh”, in part also: “like” can be deleted. Not all interviewees speak English as a native language and the benefits of better readability outweigh potential benefit from including filler words.

B. Abbreviations and shortcuts (e.g. “doesn’t” vs. “does not”, “etc.” vs “et cetera”) should be transcribed as they were originally pronounced in the interview.

C. Everyday language (e.g. “gonna” vs. “going to”) should be transcribed as they were originally pronounced in the interview

D. Language and grammar errors (e.g. “I doesn’t think so”) can be corrected at transcribers’ discretion. Minor slips of tongue can be corrected if they diminish readability.

E. Redundancies and stutters (“let us … let us talk about data sharing”) can be deleted at transcribers’ discretion if redundancies do not seem to provide additional information.

F. Pauses in the sentence or minor stutters can be marked with “…”

G. Incomprehensible passages should be marked in bold, with the timestamp of the audio file put in brackets [ ].

H. Uncertainties in the transcription process can also be marked in bold and timestamped.

I. Interviewer questions and utterances are numbered the same way as the answer numbers (I 1-X). Assertive utterances during answers (e.g. “mhm”, “yeah”, etc.) don’t need to be transcribed. Conversely, all assertive utterances by interviewees during interviewer questions are to be highlighted (in bold plus brackets), but not numbered as answers.

J. If there is more than one interviewee: Use different identification numbers and define them briefly at the beginning (e.g. Interviewee 1 = B1, interviewee 2 = B2)

K. If there are several answers to one question, e.g. in the form of a dialogue between the interviewees, please add numbering with letters, e.g.: I(1) – B(1a) – B(2a) – B(1b)

L. If there are any problems/unclarities/errors/etc.: contact the supervising associate of the Social Sciences subproject

M. Notes on data protection – anonymization: all references to the name of the interviewee are to be deleted and replaced with [name of the interviewee].

N. Notes on data protection – encryption: after exporting the file from MAXQDA as a word document, immediately assign a password according to previously discussed patterns. Permanently delete file from MAXQDA.

O. Notes on data protection – pseudonymization: the name of the file cannot provide any information about the interview. Files will be name “DATABLIC interview 1 – N”

P. Notes on data protection – transfer: Audio and text files are only transferred as encrypted files and not stored on any cloud service. If sent via official email (GCRC server), delete email from account after confirmation of reception.

Q. Notes on data protection – general: the data protection guidelines by the German Cancer Research Center apply: <https://www.dkfz.de/de/datenschutzerklaerung.html>.

R. Training and instruction: all transcriptions will be performed by trained student assistants (Social Sciences subproject and Ethics subproject), who received adequate training and instructions. Open questions and uncertainties are brought up to the supervisor (research associate of the Social Sciences subproject).

S. Software and materials: all transcriptions will be performed using official licenses of the software MAXQDA 2020. The German Cancer Research Center provides further working infrastructure, if necessary.

T. Revision: all transcripts will be double-checked by the research associate of the Social Sciences subproject.

U. Feedback: based on the revision of the interview transcripts, the research associate will provide feedback on a regular basis to help improve the transcription process. In turn, the student assistants give feedback to the interviewer using the comment function and discussions.

V. Information: the research associate of the Social Sciences subproject will provide the other subprojects with revised transcripts.

**Coding Guideline**

A. The procedure of coding the interview material follows a specific method of qualitative content analysis outlined by Kuckartz[1] as a general reference. There will be several steps of the coding process, therefore revisions of all categories will be performed at different times. The steps of the qualitative content analysis are also described in this coding guideline.

B. We start the formal process of the coding with the discussion and definition of a set of coding rules defined in this coding guideline. The codes themselves are listed and defined in the category system and the codebook.

C. There will be an initial reading of the texts, prior to the coding, to ensure sufficient preparation of the texts for the content analysis. This includes a brief summary of every interview and notes on potential highlights of the text.

D. We deductively construct a number of initial categories that are preliminary and will be tested in a first round of coding. These categories are developed and defined in a deductive process (a priori categories). The basis for the construction of the categories is the following: A) the research proposal of the Datablic project, including the general research question, the subproject’s goals, and the initial hypotheses. B) our own exploration of the relevant literature on data sharing, implementing the academic discourse on the matter. C) our own exploration of public and private funders՚ data sharing and research data policies, and the initial categories used to categorise our findings. D) the conduct and reading of the expert interviews and some early discussions to highlight noticeable findings that strongly influenced our grasp on the current practice. E) further and actualised goals and hypotheses developed by our subprojects, based on previous insights.

E. After the first rounds of discussion and coding, further categories, subcategories and aspects will be developed, and discussed out of the interview material. A priori-categories that do not show any relevant insights are removed from analysis.

F. The categories should reflect as many of the research goals of the joint project DATABLIC as possible, therefore we do not set a maximum number of categories. [At a later stage in the process, we agreed on sixteen categories].

G. Categories are not listed in a particular order. Some categories already contain possible aspects/characteristics that were determined/discussed a priori. More aspects will follow out of the coding process. If there are several relevant aspects within an aspect, it is a potential subcategory and needs its own definition at some point.

H. Since there are different priorities and subordinated research questions within the project, we allow smaller redundancies on the level of subcategories. The main categories should not overlap too much and should be clearly distinguishable. However, it is both possible and plausible that a single coding unit is coded with a plurality of codes due to minor redundancies and content overlaps (e.g., data protection might both be a legal barrier [category 2] and a need of data donors [category 9].

I. All potential hurdles, problems, ambiguities, etc. within the coding process will be discussed. Smaller issues will be discussed within the social sciences subproject, larger issues (relevant for the whole project, or the ones that could not have been solved by the social sciences subproject) will be discussed between representatives of all subprojects.

J. All coding is performed with the software MAXQDA, which was also used for the transcription of the interviews performed by the student assistants of the project. Before the coding, the social sciences research associate double-checked all transcripts in comparison to the audio-files.

K. All interviews and all material will be coded by two coders, i.e. the social sciences research associate and the social sciences student assistant. Based on our final version of the codebook, we compare and reanalyze all coding to reach consensus. Persisting disagreements will be resolved by the research associate of the ethics subproject. All categories are defined and detailed in our extended version of the category system including descriptions and applications.

L. The last part of the process, the analysis of the prepared data itself, will be performed by all subprojects.

M. Figure 1 provides an overview of the approach.

**Figure 1: Seven steps of content analysis and interview data processing**

7. Interdisciplinary content analysis

1. Initiating work on the text (Memos, highlighting important aspects, etc.)

6. Coding of all the material with differentiated category system

2. Development of main categories (mostly deductive)

Research Question

5. Development of subcategories based on the material (mostly inductive)

3. Coding of all the material gained by the interviews, using main categories

4. Aggregation of coded segments, sorted by code

**[1] Based on**: Kuckartz U (2018) Qualitative Inhaltsanalyse. Methoden, Praxis, Computerunter-stuetzung. 4th Ed., Weinheim: Beltz Juventa, p. 45. ISBN 978-3-7799-3682-4 (German)
