# Supplementary material for: Neither carrots nor sticks? Challenges surrounding data sharing from the perspective of research funding agencies—A qualitative expert interview study
Source: PLoS One. 2022 Sep 7;17(9):e0273259. doi: 10.1371/journal.pone.0273259 (PMC9451069; doi:10.1371/journal.pone.0273259)
Supplement: S3 Appendix — (DOCX) [file pone.0273259.s003.docx]

**Category System for coding and qualitative content analysis of DATABLIC expert interviews (last revision: 04/01/2022)**

**Author:** M. Anger **Contributors:** C. Wendelborn, F. Ziegler, M. Doering (German Cancer Research Center, Heidelberg), M. Tormin (Bucerius Law School, Hamburg), T. Olah (University of Heidelberg)

| **Category (number) and Subcategory (character)** |
| --- |
| **01 Incentives and support for data sharing**  a) Material incentives and support  b) Credit, recognition and reputation  c) Grant evaluation and future funding  d) Conformity with social and scientific norms and expectations  e) Collaborations, opportunities and career benefits  f) Involvement and participation in design of policies  g) Evaluation of incentives and problems for incentivizing  h) Support for data sharing  h.a) Training programs, information and workshops  h.b) Raising awareness and providing guidance  h.c) Communication and transparency  h.d) Provide infrastructure and technical support  h.e) Identification of barriers and hurdles  h.f) Funders’ support of research performing organisations (RPOs)  h.g) Support of researchers by RPOs  h.h) Make it easier for researchers in general/unspecified  -----------------------------------------------------------------------------------------------------------------------------------  **02 Barriers and hurdles for data sharing**  a) Motivational barriers  b) Technical barriers  c) Economic barriers  d) Political barriers  e) Legal barriers  f) Scientific Barriers/barriers within the scientific system  g) Ethical barriers  h) Social barriers  -----------------------------------------------------------------------------------------------------------------------------------  **03 Conflicts associated with data sharing (policies)**  a) General conflicts between funders and researchers  b) Conflicts between funders and research infrastructure  c) Conflicts between funders and other organisations  d) Conflicts about academic freedom of researchers  e) Other/non-specific conflicts  -----------------------------------------------------------------------------------------------------------------------------------  **04 Impact and benefits of data sharing policies**  a) Benefits for researchers and research communities  b) Benefits for research infrastructure (RPOS, respositories, …)  c) Benefits for funding agencies  d) Benefits for data donors  e) General positive impact or other benefits  f) Cultural Change  g) Lack of impact/negative effects  -----------------------------------------------------------------------------------------------------------------------------------  **05 Monitoring of compliance with data sharing policies**  a) Monitoring (with) data management plans (DMPs)  b) Monitoring mechanisms beyond DMPs  c) Issues with monitoring  d) Technical aspects and tools for monitoring  e) Monitoring of and by RPOs  f) Trust and social norms as substitution for monitoring  -----------------------------------------------------------------------------------------------------------------------------------  **06 Sanctions for non-compliance with data sharing policies**  a) Financial sanctions  b) Sanctions with regard to future grants and funding  c) Other kinds of sanctions  d) Concrete issues with sanctioning  e) Absence of sanctions  -----------------------------------------------------------------------------------------------------------------------------------  **07 Considerations of initiatives, networks, declarations and programs**  a) Recognition of the FAIR principles  b) Alignment with funder networks  c) Open Science declarations and strategies  d) Impact of governmental programs and initiatives  e) Other networks and programs  -----------------------------------------------------------------------------------------------------------------------------------  **08 Guidance of and participation in the development process of data sharing policies**  a) Participation/influence by researchers and research communities  b) Participation/influence by data donors  c) Participation/influence by other funding agencies  d) Participation/influence by research infrastructure  e) Participation/influence by government entities  f) Participation/influence by other entities  -----------------------------------------------------------------------------------------------------------------------------------  **09 Consideration of the rights and needs of different stakeholders**  a) Rights and needs of researchers as data producers  b) Rights and needs of researchers as secondary users of data  c) Rights and needs of data donors  d) Rights and needs of the public  e) Rights and needs of funding agencies  f) Rights and needs of research organisations  g) Rights and needs of other stakeholders/unspecified  -----------------------------------------------------------------------------------------------------------------------------------  **10 Consideration of the ethical duties and legal obligations of stakeholders**  a) Duties and obligations of funded researchers  b) Duties and obligations of other researchers  c) Duties and obligations of funding agencies  d) Duties and obligations of research organisations  e) Duties and obligations of other stakeholders/unspecified  -----------------------------------------------------------------------------------------------------------------------------------  **11 Reasonings for data sharing**  a) Maximise health benefits  b) Maximise efficiency & outputs of research/science  c) Increased reproducibility  d) Beneficial for scientists  e) Good Scientific Practice  e.a) data sharing as (a part of) good scientific practice  e.b) Research ethics & integrity  e.c) Consequences of not sharing  e.d) Scientific misconduct  f) Funded research outputs as a public good/common good  g) Other reasonings  -----------------------------------------------------------------------------------------------------------------------------------  **12 Ongoing developments, future plans and ideas concerning data sharing (policies)**  a) Future plans for monitoring and evaluation  b) Ideas about incentives and support  c) Technical developments  d) Developments regarding the own role  e) Other plans and developments  -----------------------------------------------------------------------------------------------------------------------------------  **13 Access to and storage of data: Regulations, mechanisms, and responsibilities**  a) Responsibilities around research data  b) Access and sharing mechanisms for research data  c) Storage of research data  d) Technical tools and facilitators for data access and management  e) Role of funders in facilitating access  -----------------------------------------------------------------------------------------------------------------------------------  **14 Relationship between funders and research infrastructure**  a) Communication and cooperation between funders and research infrastructures  b) Support and financing of research infrastructures by funders  c) Funders’ expectations and requirements for research infrastructures  d) Issues and problems regarding the relationship of funders and research infrastructures  -----------------------------------------------------------------------------------------------------------------------------------  **15 Kinds of data to be shared**  a) Personal data/sensitive data  b) Underlying data/supplemental data  c) Metadata  d) Going beyond data: software and materials  e) Other kinds of data/unspecified  -----------------------------------------------------------------------------------------------------------------------------------  **16 Reflections on the own role/funders’ role in the data sharing process**  a) Reflections on funders’ policies, expectations and requirements  b) Consideration of the own philosophy regarding data sharing  c) Thoughts on limits and hurdles for the own organisation  d) Relationships to other entities  e) Other reflections about the own role |

**Addendum:** For more detailed information, for example about the definition of the categories or access to the full codebook, please contact the corresponding author: [Michael.anger@dkfz-heidelberg.de](mailto:Michael.anger@dkfz-heidelberg.de) / ORCID <https://orcid.org/0000-0002-9328-510X>
